# Narcolepsy susceptibility gene *CCR3* modulates sleep-wake patterns in mice

**DOI:** 10.1371/journal.pone.0187888

**Published:** 2017-11-29

**Authors:** Hiromi Toyoda, Yoshiko Honda, Susumu Tanaka, Taku Miyagawa, Makoto Honda, Kazuki Honda, Katsushi Tokunaga, Tohru Kodama

**Affiliations:** 1 Department of Human Genetics, Graduate School of Medicine, The University of Tokyo, Tokyo, Japan; 2 Sleep Disorders Project, Department of Psychiatry and Behavioral Sciences, Tokyo Metropolitan Institute of Medical Science, Tokyo, Japan; 3 Department of Anatomy and Cell Science, Kansai Medical University, Hirakata, Japan; 4 Seiwa Hospital, Institute of Neuropsychiatry, Tokyo, Japan; Kent State University, UNITED STATES

## Abstract

Narcolepsy is caused by the loss of hypocretin (Hcrt) neurons and is associated with multiple genetic and environmental factors. Although abnormalities in immunity are suggested to be involved in the etiology of narcolepsy, no decisive mechanism has been established. We previously reported chemokine (C-C motif) receptor 3 (*CCR3*) as a novel susceptibility gene for narcolepsy. To understand the role of *CCR3* in the development of narcolepsy, we investigated sleep-wake patterns of *Ccr3* knockout (KO) mice. *Ccr3* KO mice exhibited fragmented sleep patterns in the light phase, whereas the overall sleep structure in the dark phase did not differ between *Ccr3* KO mice and wild-type (WT) littermates. Intraperitoneal injection of lipopolysaccharide (LPS) promoted wakefulness and suppressed both REM and NREM sleep in the light phase in both *Ccr3* KO and WT mice. Conversely, LPS suppressed wakefulness and promoted NREM sleep in the dark phase in both genotypes. After LPS administration, the proportion of time spent in wakefulness was higher, and the proportion of time spent in NREM sleep was lower in *Ccr3* KO compared to WT mice only in the light phase. LPS-induced changes in sleep patterns were larger in *Ccr3* KO compared to WT mice. Furthermore, we quantified the number of Hcrt neurons and found that *Ccr3* KO mice had fewer Hcrt neurons in the lateral hypothalamus compared to WT mice. We found abnormalities in sleep patterns in the resting phase and in the number of Hcrt neurons in *Ccr3* KO mice. These observations suggest a role for CCR3 in sleep-wake regulation in narcolepsy patients.

## Introduction

Narcolepsy is a sleep disorder characterized by excessive daytime sleepiness, fragmented nocturnal sleep, and abnormal rapid eye movement (REM) sleep symptoms (cataplexy, hypnagogic hallucination, and sleep paralysis). Although healthy individuals start nocturnal sleep with a non-REM (NREM) episode, narcolepsy patients show sleep-onset REM periods [1].

Narcolepsy is strongly associated with hypocretin (Hcrt) deficiency: cerebrospinal fluid hypocretin-1 levels are lower in patients relative to healthy individuals [2, 3], and hypocretin-producing neurons (Hcrt neurons) are markedly reduced in the hypothalamus of patients [4, 5]. Animal studies have clearly shown that *Hcrt* deficient mice exhibit narcolepsy-like phenotypes including behavioral arrest that is equivalent to cataplexy in human narcolepsy, sleep-onset REM periods, and fragmentation of sleep-wake episodes [6, 7]. These observations led to the simple hypothesis that narcolepsy is caused by the loss of Hcrt neurons.

Narcolepsy is associated with both genetic factors and environmental triggers, as supported by several family-based studies [8, 9]. An association of human leukocyte antigen (*HLA*)-DQB1*06:*02* with narcolepsy was initially established as a genetic factor [10, 11]. This finding led to the recognition that the study of HLA-mediated immune response in narcolepsy patients is essential for identifying the etiology. Besides the *HLA* locus, several additional narcolepsy susceptibility loci were identified by genome-wide association studies [12–18], which suggest that immune-regulating genes (*TCRA*, *P2RY11*, *TNFSF4*, *CTSH*, *TCRB*, *ZNF365*, *IL10RB-IFNAR1*, and *CCR1*/*CCR3*) and a fatty acid metabolism-related gene (*CPT1B*/*CHKB*) are involved in the development of narcolepsy. Epidemiologic studies have reported possible environmental triggers. An association with streptococcal infections was reported [19, 20]. Recently, AS03 adjuvanted vaccination against the pandemic H1N1 influenza virus or H1N1 infections themselves were reported to increase the risk of narcolepsy [21–23]. Therefore, stimulation or perturbation of the immune system can confer a risk for narcolepsy. However, how these genetic factors and environmental triggers are linked to the loss of Hcrt neurons remains unclear.

We previously reported that chemokine (C-C motif) receptor 3 (*CCR3*) is a susceptibility gene associated with narcolepsy and that its mRNA expression levels in peripheral blood are lower in narcolepsy patients [17]. CCR3 is a G protein-coupled receptor that regulates the localization of immune cells along with a gradient of chemokines secreted from inflammatory cells. The role of chemokines and their receptors in the immune system has been well established, and an increasing body of evidence suggests their roles as inflammatory mediators in the central nervous system (CNS) [24]. However, direct evidence showing that CCR is involved in sleep disorders remains limited. Therefore, our next question was whether lower expression of *CCR3* affects sleep-wake behaviors and induces sleep disorders. Furthermore, to investigate the interactive effects between genetic background (CCR3) and environmental factors, we used lipopolysaccharide (LPS), a major structural component of the outer membrane of Gram-negative bacteria, as a stimulator of the immune system. Several studies have demonstrated that LPS induces alterations in sleep-wake patterns in rodents within 24 h after administration [25–29].

In this study, we report that *Ccr3* knockout (*Ccr3* KO) mice show abnormal sleep patterns in the resting phase and a reduction in Hcrt neurons. We also found that an administration of LPS induced larger changes in sleep patterns in *Ccr3* KO compared to WT mice in the resting phase.

## Materials and methods

### Experimental animals

Male *Ccr3* KO mice on the BALB/c background (C.129S4-Ccr3<tm1Cge>/J, Stock Number 005440) (The Jackson Laboratory, Bar Harbor, ME, USA) were used for all experiments. Mice were housed in groups of 2–6 in a SPF animal facility at Tokyo Metropolitan Institute of Medical Science. All animals were housed under standard conditions (lights on at 8:00 A.M., lights off at 8:00 P.M.) with *ad libitum* access to food and water. Each mouse used in the experiments was genotyped using the following primer pairs: 5'-TGGCATTCAACACAGATGAAA-3' (*Ccr3* forward), 5'-CATGACCCCAGCTCTTTGAT-3' (*Ccr3* reverse), 5'-CTTGGGTGGAGAGGCTATTC-3' (neo forward), 5'-AGGTGAGATGACAGGAGATC-3' (neo reverse).

### Ethics statement

The experimental procedures were approved by the ethics committee on animal experiments at Tokyo Metropolitan Institute of Medical Science and conducted according to the “Guidelines for Care and Use of Laboratory Animals” of the National Institutes of Health.

### Substances

Lipopolysaccharide (LPS; *Escherichia coli* serotype O111:B4) was purchased from Sigma-Aldrich (St. Louis, MO, USA). Pyrogen-free saline (Otsuka Pharmaceutical Factory, Inc., Tokushima, Japan) was used as vehicle. LPS was reconstituted in saline and frozen until use.

### Surgery

Eight-week-old male mice were anesthetized with pentobarbital (50 mg/kg, i.p.), and implanted with cortical electroencephalogram (EEG) electrodes and nuchal electromyography (EMG) electrodes, which was modified from previous study [30]. In brief, two silver balled wires (diameter, 0.8 mm) were symmetrically placed over the cortex (1.5 mm posterior to bregma, 2.0 mm from midline) for EEG recordings, and two stainless steel wires (diameter, 0.1 mm) were embedded in the neck muscle for EMG recordings. After surgery, mice were individually habituated to the recording environment for at least 10 days in chambers at a constant temperature (25 ± 1°C) and a 12:12-h light-dark cycle with lights on from 8:00 A.M. to 8:00 P.M., corresponding to Zeitgeber time (ZT) 0–12. All mice received antibiotics immediately after surgery. All mice were monitored for signs of discomfort daily for 10 days after surgery and administered analgesic if necessary. Mice that lost 20% or more of their body weight or had markedly reduced mobility were humanely sacrificed.

### EEG/EMG recording and analyses

EEG/EMG signals derived from implanted electrodes were amplified using a multichannel amplifier (MEG-6116, Nihon Kohden, Tokyo, Japan) and recorded using VitalRecorder software (Kissei Comtec, Nagano, Japan). The signals were digitally filtered (EEG: 0.3–80 Hz; EMG: 30–300 Hz) and analyzed using SleepSign software (Kissei Comtec). EEG/EMG records were visually scored in 8-sec epochs of wakefulness (high EMG amplitude), REM sleep [low EMG amplitude, low EEG amplitude with high values in the theta band (4.0–8.0 Hz)], or NREM sleep [low EMG amplitude, high EEG amplitude with high power density in the delta band (0.5–4.0 Hz)] according to standard criteria for rodent sleep [31].

### Procedure of EEG/EMG recordings

**Fig 1:**
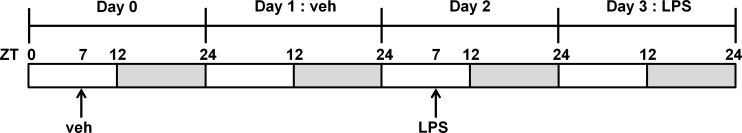
The experimental time course of EEG/EMG recordings. All WT and *Ccr3* KO mice were injected with vehicle at ZT7 on Day 0, followed by injection with 1 mg/kg LPS at ZT7 on Day 2. EEG/EMG was recorded continuously for 72 h (Days 1–3). EEG/EMG records on Day 1 and Day 3 were considered “veh” (vehicle administration) and “LPS” (after LPS administration), respectively.

Before EEG/EMG recordings were started, all WT mice (n = 8) and *Ccr3* KO mice (n = 9) were injected with vehicle on Day 0, and then recordings were started at ZT0 on Day 1. At ZT7 on Day 2, all WT mice and *Ccr3* KO mice were injected with 1 mg/kg LPS. Recordings were stopped at ZT24 on Day 3. EEG/EMG records on Day 1 and Day 3 were considered “vehicle administration” and “after LPS administration”, respectively. The experimental time course is shown in Fig 1. All injections were given intraperitoneally. Food and water were available *ad libitum* throughout the experiments.

### Immunohistochemistry

Eleven-week-old male mice (WT, n = 5; *Ccr3* KO, n = 5) were deeply anesthetized with pentobarbital in the middle of the light phase (ZT6, mice were motionless with their eyes closed) and perfused with 20 ml of cold PBS followed by 15 ml of 4% paraformaldehyde (PFA) in PBS. The brains were dissected coronally from bregma to 3.5 mm posterior to bregma using a brain slicer. Isolated brains were post-fixed in 4% PFA in PBS at 4°C for 6 h, cryo-protected in 20% sucrose in PBS at 4°C for at least 2 days, and frozen in OCT compound (Sakura Finetek, Tokyo, Japan). Floating sections were cut at 40 μm using a cryostat (Leica Biosystems, Wetzlar, Germany) and stored in 0.02% sodium azide in PBS at 4°C. Sections were incubated for 30 min in 0.1% Triton X-100 in PBS (PBST) containing 0.03%H_2_O_2_ to inactivate endogenous peroxidases and then incubated in blocking buffer (5% bovine serum albumin in PBST) at room temperature (RT) for 1 h. After blocking, sections were incubated in rabbit anti-c-Fos antibody (1:50,000, Ab-5, Calbiochem, Darmstadt, Germany) diluted in blocking buffer at 4°C for 72 h. After washes with PBST, sections were incubated in goat biotin-conjugated anti-rabbit IgG (1:1,000, Vector Laboratories, Burlingame, CA, USA) diluted in blocking buffer at RT for 2 h, followed by incubation with avidin-biotin peroxidase complex (ABC) solution (Vector Laboratories) at RT for 45 min. Tissue-bound peroxidase was visualized by incubating sections with PBS containing 0.01% 3,3'-diaminobenzidine, tetrahydrochloride (DAB) solution (Dojindo Laboratories, Kumamoto, Japan), 0.009% H_2_O_2_, and 0.5% nickel(II) chloride. For double staining, as described previously [32], sections were subsequently incubated in rabbit anti-orexin A antibody (1:2,000, H-003-30, Phoenix Pharmaceuticals, Inc., Burlingame, CA, USA) at 4°C for 24 h, followed by incubation in goat biotin-conjugated anti-rabbit IgG solution at RT for 2 h, and subsequently in ABC solution at RT for 45 min. Tissue-bound peroxidase was visualized by incubating sections with PBS containing 0.01% DAB solution and 0.009% H_2_O_2_. After washes with PBS, sections were mounted and viewed under a microscope (BZ-8100, Keyence, Osaka, Japan). The number of Hcrt neurons was counted using all (every 40 μm) hypothalamic sections between bregma -0.5 mm and bregma -2.5 mm, where Hcrt neurons exist.

### Statistical methods

The primary endpoint of this study was to determine the effect of genotype (WT, *Ccr3* KO) on each phenotype. For the time spent in each behavioral state, sleep structure, the number of states transition, and the number of cells, the two-tailed Student's *t* test was used to compare between means of two unpaired groups. A *P*-value < 0.05 was considered statistically significant.

## Results

### Sleep-wake behavior in *Ccr3* KO mice

**Fig 2:**
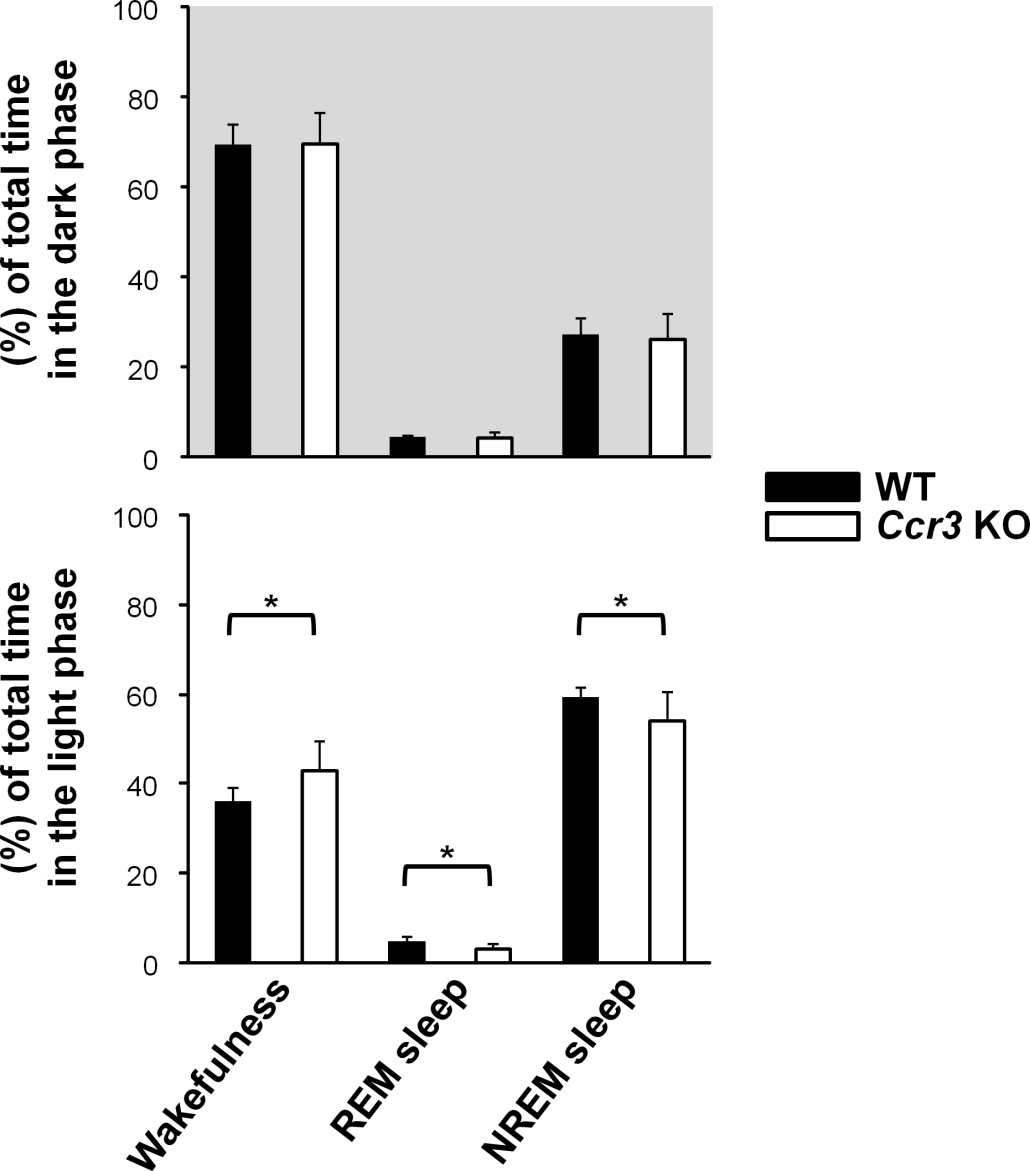
Sleep-wake behavior in *Ccr3* KO mice. Time spent in wakefulness, REM sleep, and NREM sleep was measured with EEG/EMG recording. Grey shaded areas: dark phase. Open bars indicate *Ccr3* KO mice, and black bars indicate WT mice. The two-tailed unpaired Student’s *t*-test was used to compare WT mice (n = 8) and *Ccr3* KO mice (n = 9) (*P < 0.05 and **P < 0.01). Error bars represent SE.

To investigate the role of CCR3 in the development of narcolepsy, we examined sleep-wake patterns in *Ccr3* KO mice. Both *Ccr3* KO and WT mice exhibited normal nocturnal patterns. Although the percentages of time spent in wakefulness, REM sleep, and NREM sleep in the dark phase were not significantly different between the two genotypes, these sleep-wake patterns in *Ccr3* KO mice in the light phase were significantly different compared to WT mice: the proportion of time spent in wakefulness was higher in *Ccr3* KO mice (42.9 ± 6.8% in *Ccr3* KO vs. 36.1 ± 3.1% in WT, *P* = 0.019), and the proportion of time spent in REM sleep (3.1 ± 1.2% in *Ccr*3 KO vs. 4.8 ± 1.3% in WT, *P* = 0.013) and NREM sleep (54.0 ± 6.4% in *Ccr3* KO vs. 59.1 ± 2.3% in WT, *P* = 0.047) was lower in *Ccr3* KO mice compared to WT mice (Fig 2).

**Fig 3:**
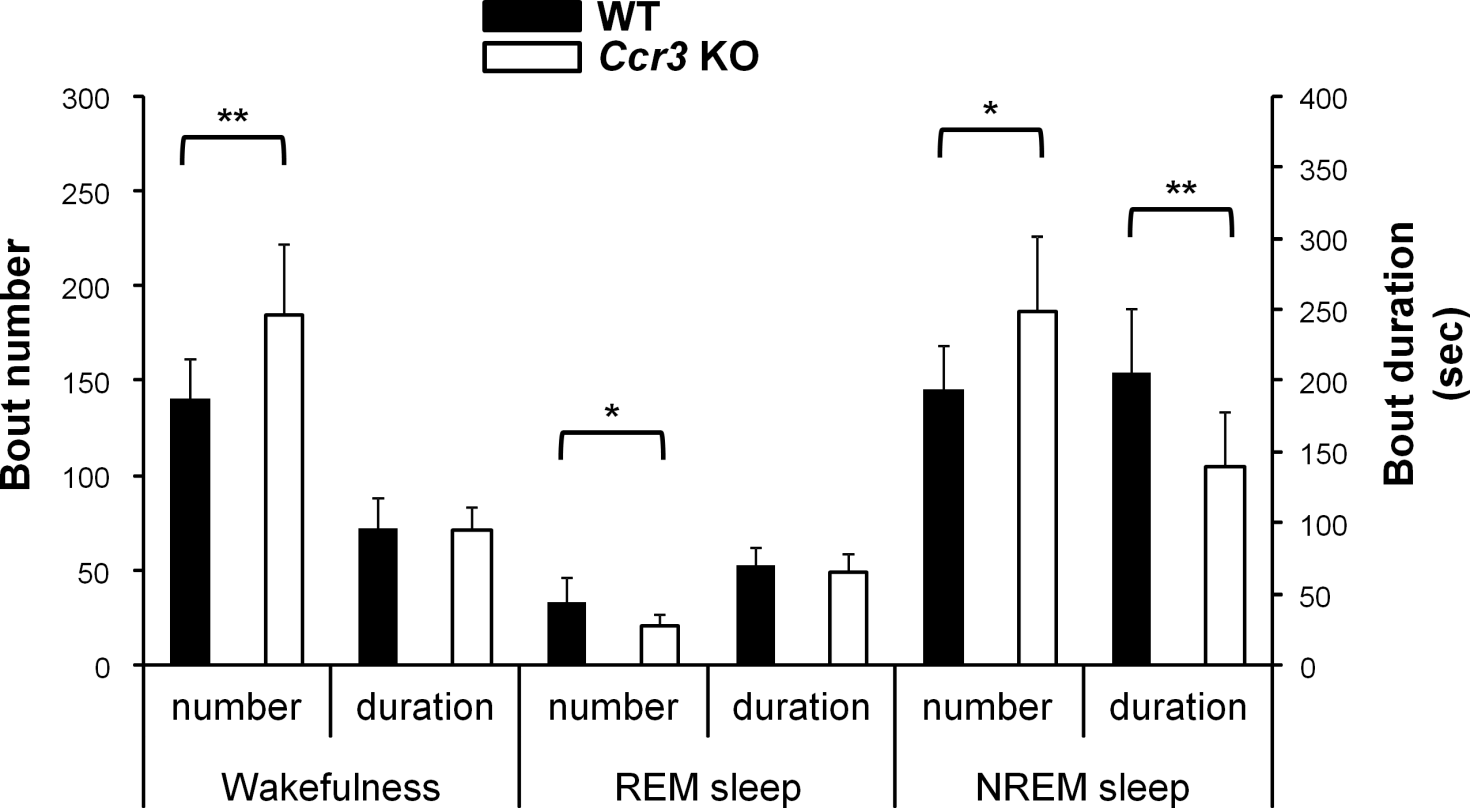
Sleep structure in *Ccr3* KO mice in the light phase. The number of bouts and the duration (sec) of each bout of each behavioral state are shown. The definitions of bars, asterisks, and error bars are provided in the Fig 2 legend.

**Fig 4:**
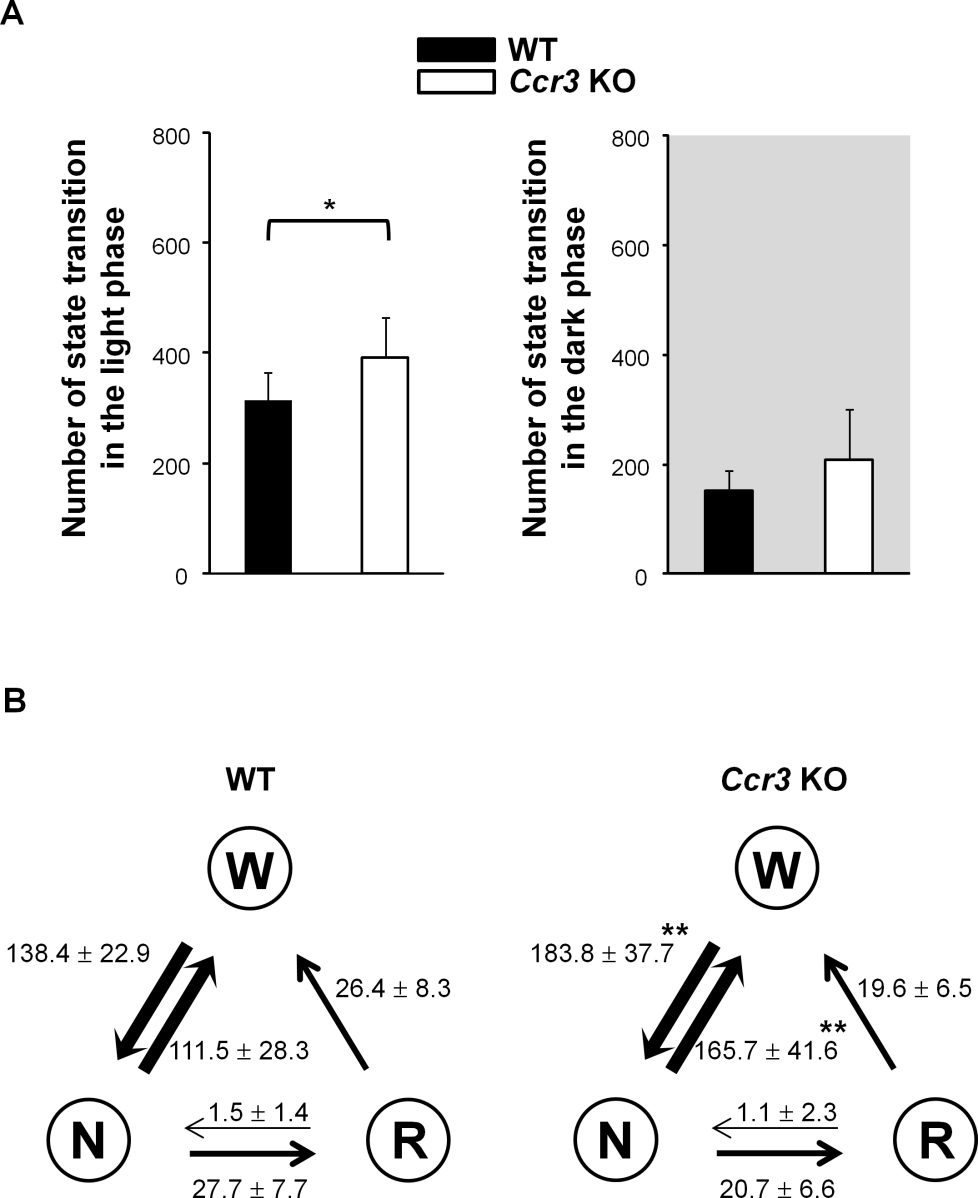
Behavioral state transition in *Ccr3* KO mice. (A) The number of behavioral state transitions was calculated. Grey shaded areas: dark phase. (B) The number of transitions (times) between each stage in the light phase was calculated. W, wakefulness; N, NREM sleep; R, REM sleep. The definitions of bars, asterisks, and error bars are provided in the Fig 2 legend.

Sleep continuity in the light phase was affected in *Ccr3* KO mice. The number of wakefulness bouts was significantly greater in *Ccr3* KO mice (184.9 ± 37.6 times in *Ccr3* KO vs. 139.1 ± 22.7 times in WT, *P* = 0.009), whereas the duration of each wakefulness bout did not differ from WT mice (Fig 3). The number of REM sleep bouts was fewer in *Ccr3* KO mice (20.7 ± 6.6 times in *Ccr3* KO vs. 32.0 ± 14.1 times in WT, *P* = 0.047). Interestingly, the number of NREM sleep bouts was significantly greater in *Ccr3* KO mice (186.6 ± 38.8 times in *Ccr3* KO vs. 143.8 ± 25.0 times in WT, *P* = 0.018), and the duration of each NREM bout in *Ccr3* KO mice was significantly shorter than that in WT mice (139.3 ± 37.6 sec in *Ccr3* KO vs. 203.4 ± 47.6 sec in WT, *P* = 0.007) (Fig 3). These findings indicate that the increased number of NREM bouts and the shorter NREM sleep duration in *Ccr3* KO mice are due to the increased number of wakefulness bouts, which suggests fragmented sleep. The calculated number of transitions between different behavioral states in the light phase was greater in *Ccr3* KO mice (391.4 ± 74.2 times in *Ccr3* KO vs. 313.9 ± 49.9 times in WT, *P* = 0.025) (Fig 4A). The number of transition between wakefulness and NREM sleep in the light phase was significantly increased in *Ccr3* KO mice compared to WT mice (wakefulness to NREM sleep, 183.8 ± 37.7 times in *Ccr3* KO vs. 138.4 ± 22.9 times in WT, *P* = 0.001; NREM sleep to wakefulness, 165.7 ± 41.6 times in *Ccr3* KO vs. 111.5 ± 28.3 times in WT, *P* = 0.007) (Fig 4B). The number of transition among other states was not significantly different between the two genotypes.

### The effects of LPS on sleep-wake behavior in *Ccr3* KO mice

**Fig 5:**
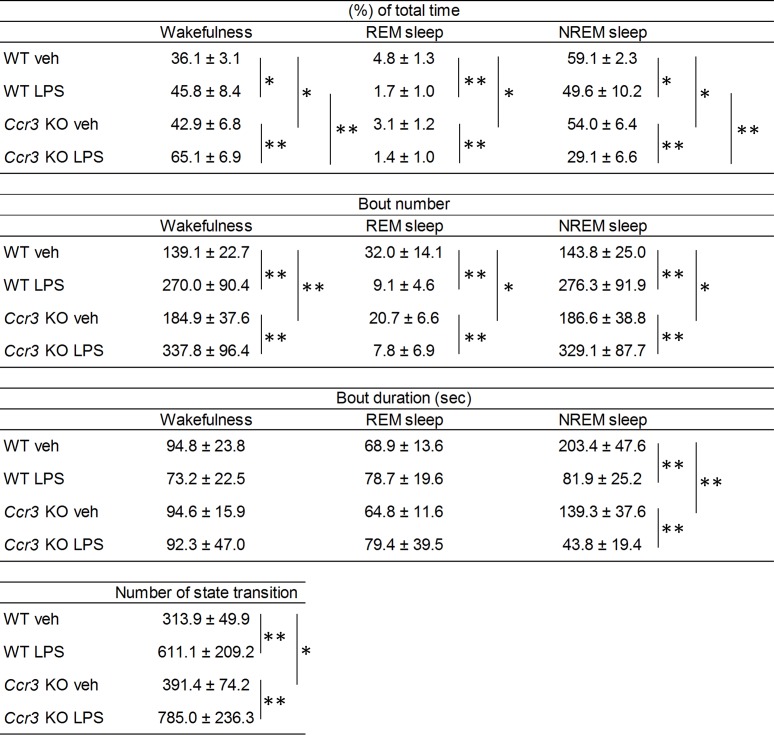
Sleep structure in the light phase. Values are mean ± SE of 8 WT and 9 *Ccr3* KO mice. The two-tailed unpaired Student’s *t*-test was used to compare means between two unpaired groups. *P < 0.05; **P < 0.01.

**Fig 6:**
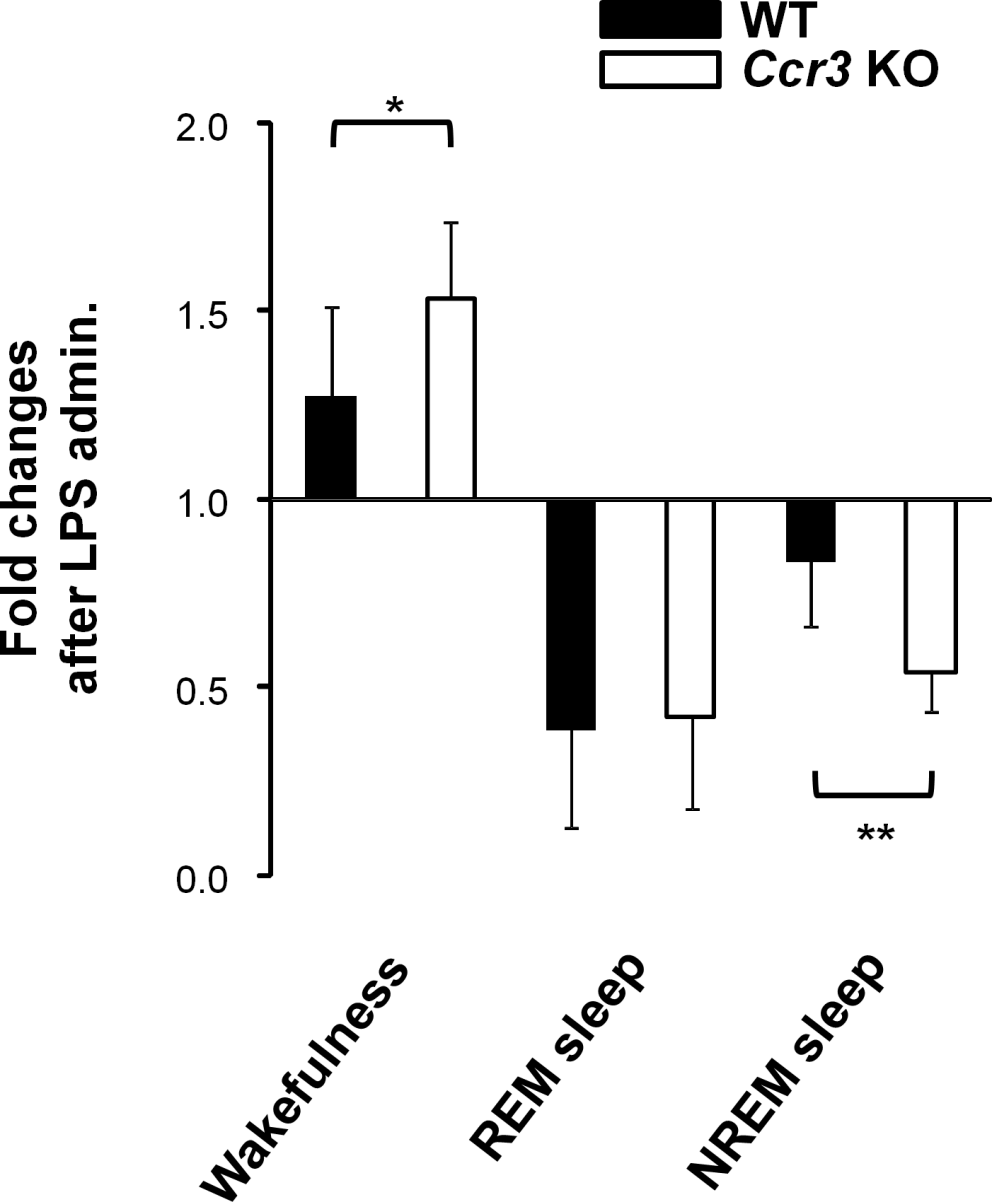
Changes induced by LPS administration. Fold changes in time spent in each state in the light phase after LPS administration are shown (1.0 = vehicle administration). The definitions of bars, asterisks, and error bars are provided in the Fig 2 legend.

We next examined whether an environmental trigger can affect sleep-wake patterns in *Ccr3* KO mice using intraperitoneal administration of LPS. LPS administration significantly increased wakefulness (45.8 ± 8.4% in WT LPS vs. 36.1 ± 3.1% in WT veh, *P* = 0.014; 65.1 ± 6.9% in *Ccr3* KO LPS vs. 42.9 ± 6.8% in *Ccr3* KO veh, *P* = 3.7 × 10^−6^), and decreased NREM sleep (49.6 ± 10.2% in WT LPS vs. 59.1 ± 2.3% in WT veh, *P* = 0.033; 29.1 ± 6.6% in *Ccr3* KO LPS vs. 54.0 ± 6.4% in *Ccr3* KO veh, *P* = 4.9 × 10^−7^) in the light phase in both *Ccr3* KO and WT mice (Fig 5). The number of state transitions in the light phase were increased after LPS administration in both genotypes (611.1 ± 209.2 times in WT LPS vs. 313.9 ± 49.9 times in WT veh, *P* = 0.005; 785.0 ± 236.3 times in *Ccr3* KO LPS vs. 391.4 ± 74.2 times in *Ccr3* KO veh, *P* = 0.0009) (Fig 5). Similar to before LPS administration, the proportion of time spent in wakefulness was higher (65.1 ± 6.9% in *Ccr3* KO LPS vs. 45.8 ± 8.4% in WT LPS, *P* = 0.0001), and the proportion of time spent in NREM sleep was lower (29.1 ± 6.6% in *Ccr3* KO LPS vs. 49.6 ± 10.2% in WT LPS, *P* = 0.0002) in *Ccr3* KO mice compared to WT mice (Fig 5). However, the changes induced by LPS administration were significantly larger in *Ccr3* KO mice compared to WT mice in the light phase (wakefulness, 1.5 ± 0.2-fold change in *Ccr3* KO vs. 1.3 ± 0.3-fold change in WT, *P* = 0.032; NREM sleep, 0.5 ± 0.1-fold change in *Ccr3* KO vs. 0.8 ± 0.2-fold change in WT, *P* = 0.0007) (Fig 6). We observed a tendency in which LPS induced an increased number of state transitions in *Ccr3* KO mice compared to WT mice (Fig 5).

**Fig 7:**
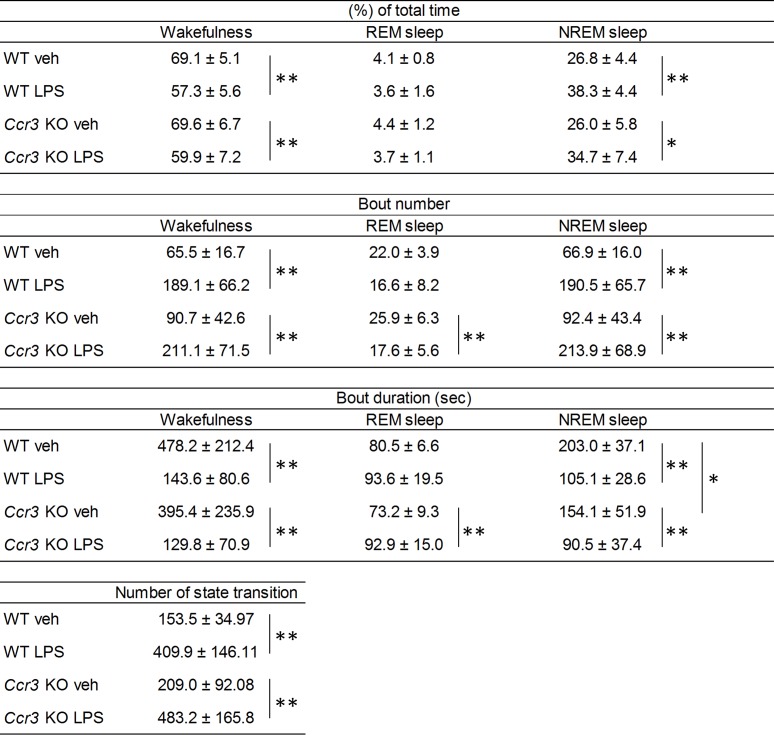
Sleep structure in the dark phase. Values are mean ± SE of 8 WT and 9 *Ccr3* KO mice. The two-tailed unpaired Student’s *t*-test was used to compare means between two unpaired groups. *P < 0.05; **P < 0.01.

In contrast to the light phase, LPS administration significantly decreased the proportion of time spent in wakefulness and increased the proportion spent in NREM sleep in the dark phase in both *Ccr3* KO and WT mice, but similar to before LPS administration, the proportion of time spent in each behavioral state in the dark phase after LPS administration was not different between the two genotypes (Fig 7).

### Decreased Hcrt neurons in *Ccr3* KO mice

**Fig 8:**
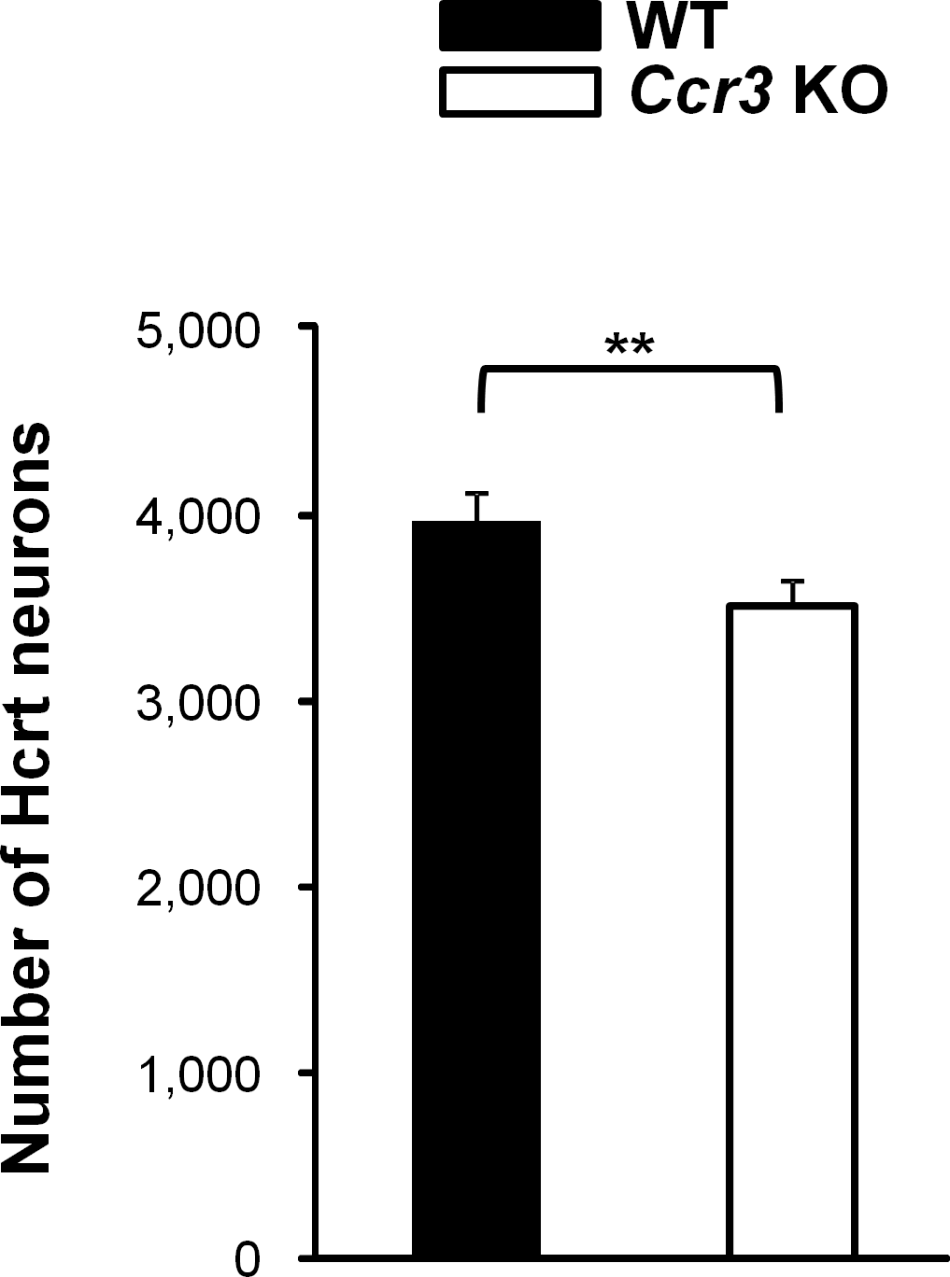
Decreased Hcrt neurons in *Ccr3* KO mice. The number of Hcrt neurons in *Ccr3* KO mice was quantified. The two-tailed unpaired Student’s *t*-test was used to compare WT mice (n = 5) and *Ccr3* KO mice (n = 5) (**P < 0.01). Error bars represent SE.

Since *Hcrt* deficient mice reportedly exhibit fragmented sleep patterns [6, 7, 33], we quantified the number of Hcrt neurons in *Ccr3* KO mice. *Ccr3* KO mice had significantly fewer HCRT immunoreactive cells compared to WT mice (3497.4 ± 135.2 in *Ccr3* KO vs. 3963.8 ± 149.4 in WT, *P* = 0.0009) (Fig 8). No significant genotype-dependent differences were observed in the number of FOS-immunoreactive Hcrt neurons (S1A and S1B Fig). We also quantified prepro-*Hcrt* mRNA levels and hypocretin-1 peptide levels in the hypothalamus (S1 Methods). Neither the mRNA nor the peptide levels were significantly different between the two genotypes (S2 and S3 Figs).

## Discussion

In the present study, we showed that *Ccr3* KO mice exhibit fragmented sleep patterns in the light phase (Figs 2–4). To our knowledge, this is the first report showing that CCR3 is involved in the sleep regulation. We also observed a slight but significant decrease in the number of Hcrt neurons (Fig 8).

The sleep architecture of *Hcrt* deficient mice has been thoroughly investigated using prepro-*Hcrt* KO mice [6] and Hcrt neuron-ablated (*Hcrt/ataxin3*) mice [7]. These mice exhibit cataplexy, and direct transitions to REM sleep from wakefulness were observed in both studies. Fragmentation of wakefulness in the dark phase was also demonstrated: a shared phenotype of a shorter duration of wakefulness episodes was observed in *Hcrt* deficient mice compared to WT mice [6, 7]. A recent study using conditional Hcrt neuron-ablated mice, in which Hcrt neurons can be manipulated to degenerate during adulthood, reported that fragmented sleep-wake cycles occur after loss of 80% of Hcrt neurons, and cataplexy is detected after loss of 95% of Hcrt neurons [33]. They also showed that the transition frequency between sleep-wake states is significantly increased in *Hcrt* deficient mice only in the dark phase, and not in the light phase. In our study, the number of transitions between behavioral states was increased in *Ccr3* KO mice only in the light phase, although a similar tendency was observed in the dark phase (Fig 4A). Concurrent with this observation, the differences in sleep structure between *Ccr3* KO and WT mice were observed only in the light phase, except for the shorter duration of NREM sleep bouts in *Ccr3* KO mice in the dark phase (Figs 5 and 7). The phenotypes of *Ccr3* KO mice were different from those of *Hcrt* deficient mice; however, the fragmented sleep-wake patterns of *Ccr3* KO mice in the light phase are suggestive of fragmented nocturnal sleep in human narcolepsy.

Our previous study reported that impaired *CCR3* function is a risk for narcolepsy [17]; yet, a defect only in *CCR3* is not sufficient for the disease development because polygenic risks for narcolepsy have been estimated to explain 58.1% of narcolepsy onset in the context of the contribution of common variants [34]. Therefore, we sought to address whether some triggers besides genetic factors can affect sleep-wake patterns in *Ccr3* KO mice. Administration of LPS increased wakefulness and decreased NREM sleep in both *Ccr3* KO and WT mice in the light phase (Fig 5), but the changes induced by LPS administration were significantly larger in *Ccr3* KO mice compared to WT mice in the light phase (Fig 6). We also found a tendency in which LPS administration induced more fragmented sleep patterns in *Ccr3* KO mice in the light phase compared to WT mice (Fig 5). In contrast to the light phase, no significant genotype-dependent differences were observed in the phenotypes during the dark phase regardless of LPS administration. The sleep-wake state is regulated by a cluster of neurons in the hypothalamus. Sleep-promoting neurons located in the ventrolateral preoptic nucleus (VLPO) are dominant in the resting phase, whereas wake-promoting neurons in the tuberomammillary nucleus and in the lateral hypothalamus are active during vigilance [35]. Our results suggest that CCR3 promotes NREM sleep in the sleep-promoting system that is dominant in the resting phase. Accordingly, the resting-phase sleep state of *Ccr3* KO mice may be easily unstabilized by external stimuli (LPS in this study).

The effects of LPS administration on sleep have been examined in several studies [25–29]. In the dark phase, a shared phenotype of an increased NREM sleep is observed regardless of experimental condition. LPS also reportedly induces fragmented sleep [25, 29]: the increased number of NREM bouts and the shorter NREM sleep duration. Our study replicated these previous findings (Fig 7), suggesting the validity of our results. The result of no genotype-dependent differences in the phenotypes indicates that CCR3 plays a minor role in the arousal state.

The LPS-induced phenotypes observed in the light phase are reportedly different depending on the timing of administration [28]: administration at dark onset partially suppresses REM sleep with no effects on NREM sleep, but administration at light onset completely suppresses REM sleep and promotes NREM sleep. In our study, LPS administration decreased NREM sleep in the light phase. One possible explanation is that LPS administration induces inflammatory hyperalgesia [36] that might result in acute suppression of NREM sleep. We suppose that *Ccr3* KO mice are more vulnerable to such disruption in regulation of NREM sleep in the light phase compared to WT mice.

Based on these findings, we hypothesize that CCR3 promotes NREM sleep in the resting phase, and thus CCR3 deficiency is more likely to cause NREM sleep dysregulation after exposure to environmental stimuli that results in fragmented sleep. More studies are needed to understand how CCR3 contributes to the regulation of NREM sleep. For instance, it is crucial to examine whether *Ccr3* is expressed and functions in the VLPO. Limitation of our study is that we used only LPS. An investigation using other immune stimulators such as viral single/double-stranded DNA/RNA is essential for understanding the interactive contribution of CCR3 and environmental factors to the narcolepsy development.

We found that *Ccr3* KO mice have ~10% fewer Hcrt neurons compared to WT mice. This small decrease alone is not expected to directly induce fragmented sleep because loss of 80% of Hcrt neurons is necessary to cause fragmented sleep-wake cycles that result in narcolepsy [33]. Furthermore, *Ccr3* KO mice did not show cataplexy-like behaviors or sleep-onset REM periods, which are typical phenotypes of narcolepsy. According to Tabuchi et al., cataplexy-like behaviors in *Hcrt* deficient mice are strongly dependent on the Hcrt system. Given these previous reports and our results, fragmented sleep-wake patterns in *Ccr3* KO mice in the light phase may be mediated by alternative unknown mechanisms other than the Hcrt system. However, CCR3 may be necessary for survival of Hcrt neurons. Links between neuronal cell survival and CCR3 have been described: *Ccr3*-expressing CD4+ T cells are necessary for facial motoneuron survival after facial nerve axotomy [37], whereas CCR3 promotes neuronal cell death in a post-infarction mouse model [38]. These reports suggest that the role of CCR3 in neuronal survival is different depending on the context of disease. As genetic and epidemiologic studies have suggested, a loss of Hcrt neurons in patients with narcolepsy is considered to be caused by an autoimmune-mediated process [39]. Multiple studies using sera and cerebrospinal fluids have revealed abnormalities in the levels of cytokines in patients with narcolepsy [40–43]. Despite evidence indicating abnormalities in immunity, little is known about the state of the immune system during the process of degeneration of Hcrt neurons in narcolepsy patients. A recent report suggested that Th1-related cytokines tend to be increased in narcolepsy patients very close to disease onset [44]. The cytokines that are elevated include chemokine (C-C motif) ligand 5 (CCL5), a ligand for CCR3. Another study showed that intramuscular injection of AS03 alone induces transient expression of chemokines including CCL5 in the injection site of mice [45]. Given that AS03 adjuvanted vaccinations increase the risk for narcolepsy, we speculate that elevated chemokines may mediate the immune response that results in the increased risk of narcolepsy; meanwhile, the CCL5 receptor, CCR3, plays a protective role in Hcrt neuronal survival.

In conclusion, we propose that impaired function of CCR3 causes fragmented sleep that can contribute to vulnerability to developing narcolepsy in an inflammatory context induced by environmental triggers. Understanding the context of narcolepsy onset using animal models of immune-mediated degeneration of Hcrt neurons will be crucial.

## Supporting information

S1 Fig FOS-IR Hcrt neurons in the hypothalamic region. (A) The number of FOS-IR Hcrt neurons and (B) the ratio of FOS-IR neurons to Hcrt IR neurons are shown. *The two-tailed unpaired Student’s *t*-test was used to compare WT mice (n = 5) and *Ccr3* KO mice (n = 5). Error bars represent SE. ns: not significant. IR: immunoreactive.
(TIF)

S2 Fig Analysis of *Hcrt* expression in the hypothalamic region. *The two-tailed unpaired Student’s *t*-test was used to compare WT mice (n = 3) and *Ccr3* KO mice (n = 3). Error bars represent SE. ns: not significant.
(TIF)

S3 Fig Analysis of hypocretin-1 content in the hypothalamic region. *The two-tailed unpaired Student’s *t*-test was used to compare WT mice (n = 4) and *Ccr3* KO mice (n = 4). Error bars represent SE. ns: not significant.
(TIF)

S1 Methods Methods for quantitative RT-PCR and hypocretin-1 ELISA. (DOCX)
