# Supplementary figures and images for: Narcolepsy susceptibility gene CCR3 modulates sleep-wake patterns in mice
Source: PLoS One. 2017 Nov 29;12(11):e0187888. doi: 10.1371/journal.pone.0187888 (PMC5706730; doi:10.1371/journal.pone.0187888)

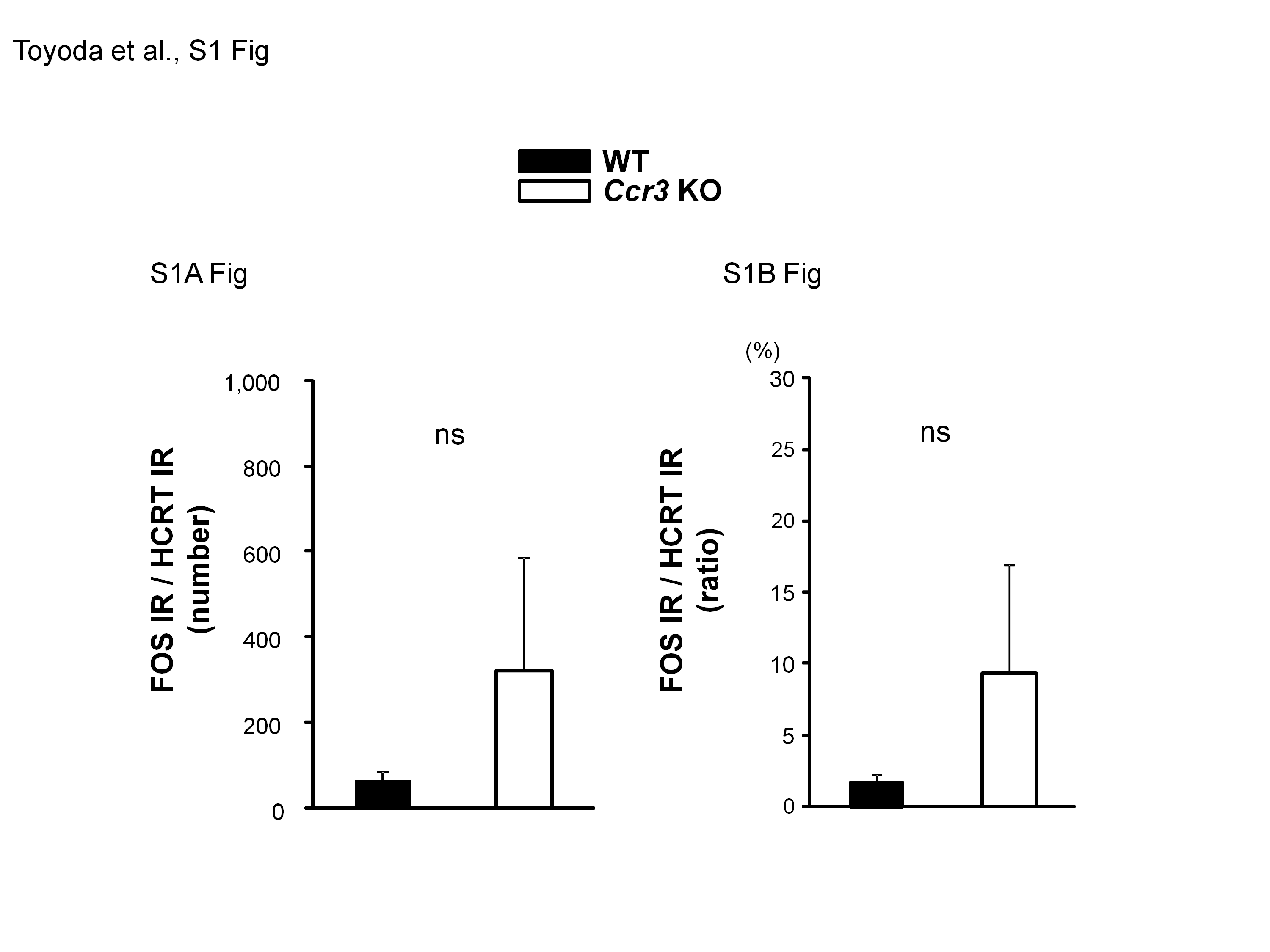

Supplement: S1 Fig — (A) The number of FOS-IR Hcrt neurons and (B) the ratio of FOS-IR neurons to Hcrt IR neurons are shown. *The two-tailed unpaired Student’s t-test was used to compare WT mice (n = 5) and Ccr3 KO mice (n = 5). Error bars represent SE. ns: not significant. IR: immunoreactive. (TIF) [file pone.0187888.s001.tif]

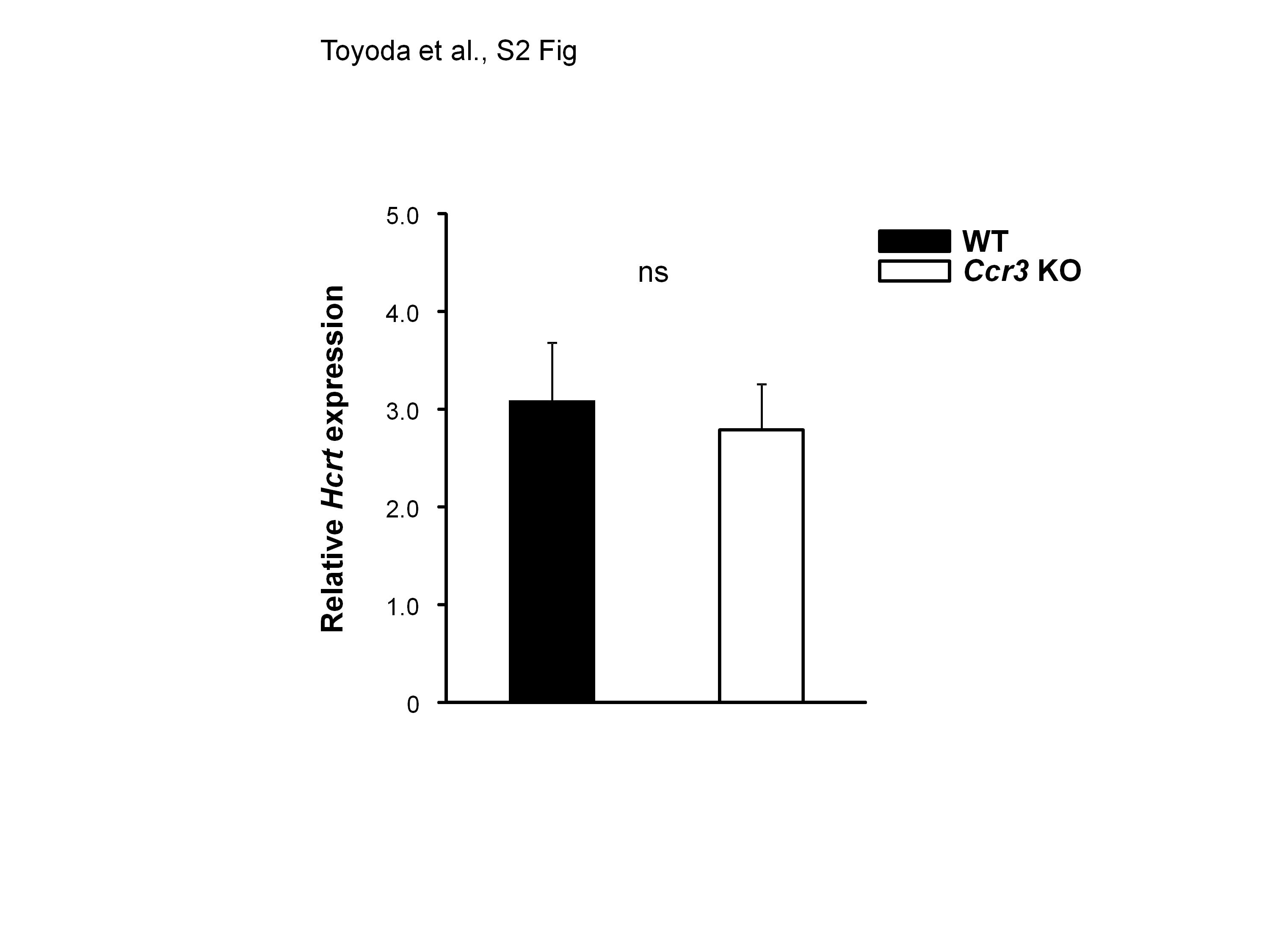

Supplement: S2 Fig — *The two-tailed unpaired Student’s t-test was used to compare WT mice (n = 3) and Ccr3 KO mice (n = 3). Error bars represent SE. ns: not significant. (TIF) [file pone.0187888.s002.tif]

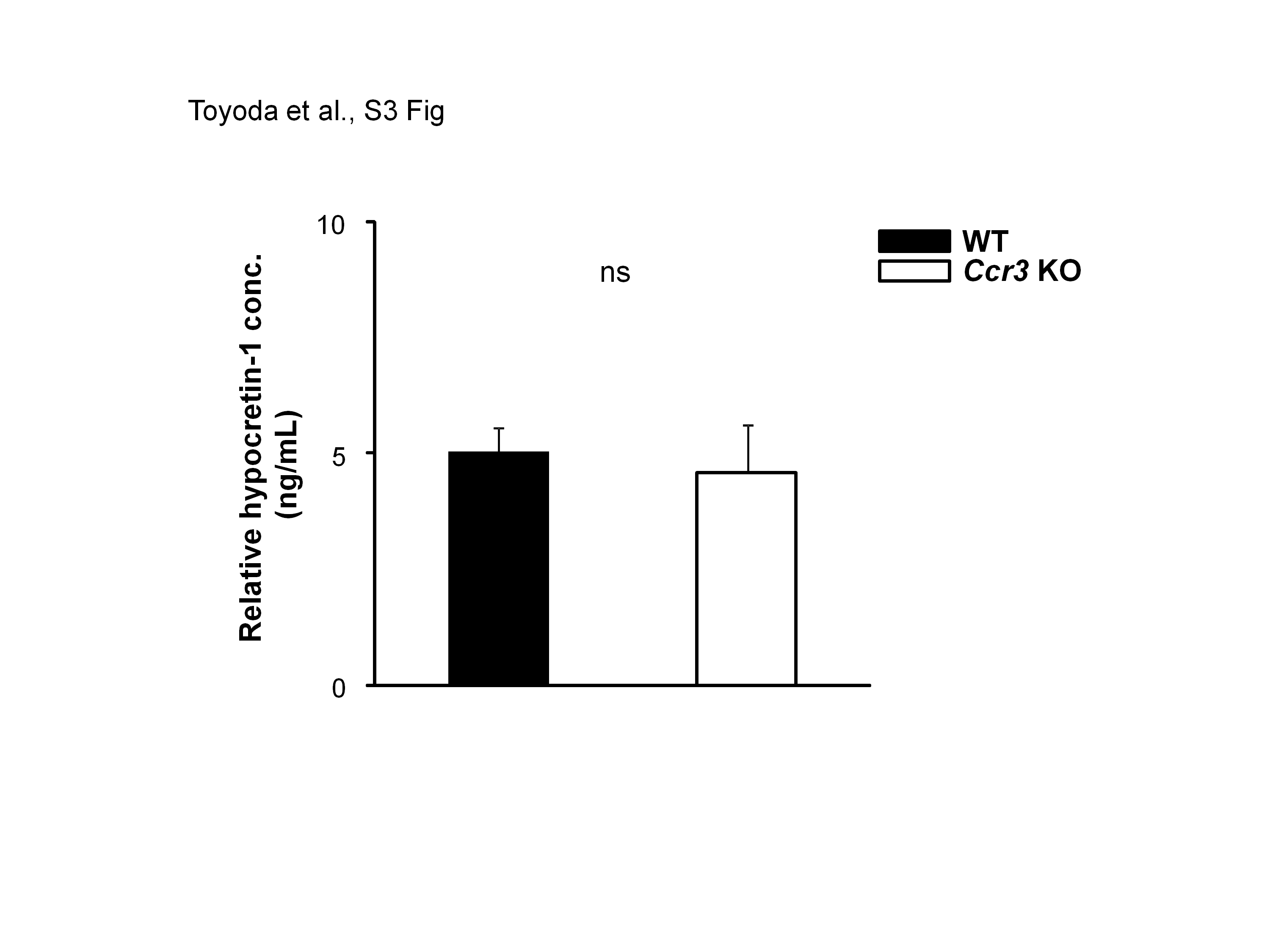

Supplement: S3 Fig — *The two-tailed unpaired Student’s t-test was used to compare WT mice (n = 4) and Ccr3 KO mice (n = 4). Error bars represent SE. ns: not significant. (TIF) [file pone.0187888.s003.tif]
