# Supplementary material for: Narcolepsy susceptibility gene CCR3 modulates sleep-wake patterns in mice
Source: PLoS One. 2017 Nov 29;12(11):e0187888. doi: 10.1371/journal.pone.0187888 (PMC5706730; doi:10.1371/journal.pone.0187888)
Supplement: S1 Methods — (DOCX) [file pone.0187888.s004.docx]

**S1 Methods**

**Quantitative RT-PCR**

The brains were dissected coronally from bregma to 3.5 mm posterior to bregma using a brain slicer at ZT5. Hypothalami were removed, immediately snap-frozen, and stored at −80ºC until RNA extraction. Total RNA was isolated from each hypothalamus using RNeasy Lipid Tissue Mini Kit (Qiagen, Venlo, The Netherlands). cDNA synthesis was performed with the PrimeScript RT reagent Kit with gDNA Eraser (Takara Bio, Shiga, Japan) according to the manufacturer’s instructions. SYBR Premix Ex Taq (Takara Bio) and the ABI 7300 Real-Time PCR system (Life Technologies, Gaithersburg, MD, USA) were used to perform quantitative PCR in duplicate. The relative standard curve method was used to measure the expression level of each gene. Each mRNA level was normalized to the *Rps18* mRNA level. The following primer pairs were used for amplification: 5'-CTCCAGGCACCATGAACTTT-3' (*Hcrt* forward), 5'-GGGATGTGGCTCTAGCTCTG-3' (*Hcrt* reverse). The mouse *Rps18* primers were purchased from Takara Bio.

**Hypocretin-1 ELISA**

The hypothalamic region was dissected as described above. Each frozen hypothalamus was boiled in water for 10 min. After cooling on ice, 17 N acetic acid and 12 N HCl were added to make final concentrations of 1 M and 20 mM, respectively (20-fold volume for each dissected hypothalamus). Subsequently, the hypothalamus was homogenized by sonication in an ice-cold bath for 30 sec-1 min, followed by centrifugation at 20,000 ×*g* for 15 min at 4°C. The supernatant was filtered through a 0.45 μm PVDF syringe filter (Millipore, Darmstadt, Germany), and then subjected to ELISA using a commercially available kit (EK-003-30, Phoenix Pharmaceuticals Inc., Burlingame, CA, USA). The supernatant was diluted 1:10 in 1× assay buffer supplied in the kit. ELISA was performed in duplicate according to the manufacturer’s instructions. The total protein concentration in the supernatant was estimated using the BCA assay (Thermo Fisher Scientific, Waltham, MA, USA), and the hypocretin-1 concentration was normalized to the total protein concentration.
